# Gut Microbiome Dysbiosis in Patients with Endometrial Cancer vs. Healthy Controls Based on 16S rRNA Gene Sequencing

**DOI:** 10.1007/s00284-023-03361-6

**Published:** 2023-06-09

**Authors:** Yue Li, Geng Liu, Runqi Gong, Yong Xi

**Affiliations:** 1 Department of Obstetrics and Gynecology, Dalian Municipal Women and Children’s Medical Center (Group), No.1, Dunhuang Road, Shahekou District, Dalian, Liaoning 116033 P.R. China; 2 Department of Obstetrics and Gynecology, Liaoning Provincial Hospital for women and children, Shenyang, Liaoning 110004 P.R. China

## Abstract

Metabolic diseases like obesity, diabetes, and hypertension are considered major risk factors associated with endometrial cancer. Considering that an imbalance in the gut microbiome may lead to metabolic alterations, we hypothesized that alteration in the gut microbioma might be an indirect factor in the development of endometrial cancer. Our aim was to profile the gut microbiota of patients with endometrial cancer compared with healthy controls in this study. Thus, we used 16S rRNA high-throughput gene sequencing on the Illumina NovaSeq platform to profile microbial communities. Fecal samples were collected from 33 endometrial cancer patients (EC group) and 32 healthy controls (N group) between February 2021 and July 2021. The total numbers of operational taxonomic units (OTUs) in the N and EC groups were 28,537 and 18,465, respectively, while the number of OTUs shared by the two groups was 4771. This study was the first to report that the alpha diversity of the gut microbiota was significantly reduced in endometrial cancer patients vs. healthy controls. Also, there was a significant difference in the distribution of microbiome between the two groups: the abundance of *Firmicutes*, *Clostridia*, *Clostridiales*, *Ruminococcaceae, Faecalibacterium*, and *Gemmiger_formicis* decreased, while that of *Proteobacteria*, *Gammaproteobacteria*, *Enterobacteriales*, *Enterobacteriaceae* and *Shigella* increased significantly in the EC group vs. healthy controls (all *p < 0.05*). The predominant intestinal microbiota of the endometrial cancer patients was *Proteobacteria*, *Gammaproteobacteria*, *Enterobacteriales*, *Enterobacteriaceae*, and *Shigella*. These results imply that adjusting the composition of the gut microbiota and maintaining microbiota homeostasis may be an effective strategy for preventing and treating endometrial cancer.

**Supplementary Information:**

The online version contains supplementary material available at 10.1007/s00284-023-03361-6.

## Introduction

Endometrial cancer is the most common gynecological malignant tumor in developed countries [1]. Recently, its incidence and mortality rates have been increasing [2]; in 2021, 66,570 new cases and 12,940 endometrial cancer deaths were reported in the United States [3]. Histologically, endometrial cancer can be divided into estrogen-dependent (type I) and nonestrogen-dependent (type II), with the former one being more common and accounting for 80-90% of all cases [4]. Type I endometrial cancers are often associated with metabolic syndrome (MS) (e.g., obesity, hypertension, diabetes) [5–7]; other risk factors include menopausal delay or anovulatory diseases, functional ovarian tumors, long-term single estrogen treatment, etc. [8, 9]. However, there is still no clear conclusion on the mechanisms and causes of endometrial cancer.

According to recent studies, the microbiome may also be an indirect risk factor for endometrial cancer [10]. The human gut microbiome is characterized by various microorganisms (mainly Firmicutes, Bacteroidetes, Proteobacteria, and Actinobacteria) with complex functions. They coexist with the host in a symbiotic manner and participate in a series of physiological processes that are critical to the health of the host, including metabolism, energy homeostasis, immune response regulation, neurobehavioral development, maintenance of intestinal mucosal epithelial integrity and homeostasis of the internal environment [11, 12]. Alterations in gut microbiome abundance have been associated with the occurrence of various human diseases, such as intestinal inflammation, lung disease, and colorectal cancer [13–15]. Moreover, in recent years, increasing evidence has shown that the intestinal microbiome and its metabolites have a critical role in the occurrence and progression of chronic metabolic diseases, including obesity, diabetes, fatty liver disease, atherosclerosis, hypertension, etc. [16, 17]. Plottel et al. reported that the human gut microbiome, such as *Bifidobacterium, Clostridium* and *Lactobacillus* was involved in host estrogen metabolism by secreting β-glucuronidase; the disturbance of the intestinal microbiota can lead to elevated estrogen levels and the occurrence of estrogen-related diseases [18]. Other studies found an association between intestinal microbes and the pathogenesis of polycystic ovary syndrome (PCOS) [19]. In addition, Zhou et al. discovered that intestinal microbiota is associated with the serum hormone levels and clinical characteristics of PCOS patients [20]. Considering that these metabolic diseases are relatively established risk factors for endometrial cancer, it is possible that gut microbioma may be an indirect factor in the development of endometrial cancer. Thus, in this study, we used 16S rRNA high-throughput gene sequencing on the Illumina NovaSeq platform to profile microbial communities in endometrial cancer patients and healthy controls. Possible mechanisms of intestinal microbiota affecting the etiology of endometrial cancer were also analyzed.

## Materials and Methods

### Participants

A total of 33 endometrial cancer patients (EC group) and 32 healthy controls (N group) were recruited from Dalian Municipal Women and Children’s Medical Center Hospital between February 2021 and July 2021. The inclusion criteria for the N group were: inpatients admitted to our hospital and scheduled to undergo diagnostic curettage (no endometrial lesions were found). The inclusion criteria for the EC group were: patients pathologically diagnosed (according to the International Federation of Gynecology and Obstetrics (FIGO), 2009 standard) with endometrial cancer (type I endometrial cancer) who received surgical treatment, and their histological type was endometrioid cancer.

The exclusion criteria were: (1) those who have used antibiotics, probiotics, or proton pump inhibitors within 1 month before enrollment; (2) previously suffered from inflammatory bowel disease or other diseases affecting the gut microbiota; (3) with abnormal intestinal symptoms (abdominal pain, diarrhea, constipation, etc.); (4) with malignant tumors of other organs; and (5) with severe mental illness or other conditions that precluded them from completing or participating in the study.

The clinicopathological data were complete, and all histological sections were reviewed by pathologists at our hospital.

### Sample Collection, DNA Extraction, and 16S rRNA Gene Sequencing

In a clean environment, sterile gloves, sterile forceps, and sterilized 2.0 ml centrifuge tubes were used to collect fresh feces from the patients. The sampling volume of a single sample was approximately 1.0 g/tube, and the sample was frozen at − 80 °C for further use. To ensure the progress of the experiment, 3 replicates of each sample were taken for backup. Fecal genomic DNA was extracted using an Omega soil DNA Kit (m5635-02) (Omega Bio-Tek, Norcross, GA, USA) following the manufacturer’s instructions and temporarily stored at -20 °C for standby. The diversity composition spectrum of the gut microbioma samples was sequenced and analyzed using Illumina NovaSeq next-generation sequencing platforms (Illumina, Inc, San Diego, CA, USA). The bacterial 16 S rRNA gene double V-variable region (V3V4) was amplified by using the forward primer 338 F (5’-ACTCCTACGGGAGGCAGCA-3’) and reversed primer 806R (5’-GGACTACHVGGGTWTCTAAT-3’) (Personalbio Technology, Shanghai, China). The PCR mixture included 5 µl of buffer (5×), 0.25 µl of Fast Pfu DNA Polymerase (5 U/µl), 2 µl of dNTPs, 1 µl of DNA template,1 µl of each primer, and 14.75 µl of ddH2O. Parameters of the PCR thermal cycle were as follows: 25 cycles of predenaturation at 98 °C for 5 min, denaturation at 98 °C for 30 s, annealing at 52 °C for 30 s, extension at 72 °C for 45 s, and final extension at 72 °C for 5 min. The Illumina NovaSeq sequencing library was prepared and sequenced using the amplified product as a template.

The raw 16S rRNA gene sequences of 65 fecal samples were deposited in NCBI Sequence Read Archive (SRA) database with the accession number PRJNA897842.

### Bioinformatics Analysis

The sequence data analysis was performed using QIIME 2 (Version 2019.4) [21] and R packages (Version 3.2.0). After denoising and clustering the obtained sequences, operational taxonomic units (OTUs) were determined according to similarity ≥97%. QIIME2 was used to draw the rarefaction curve and calculate the number of OTUs and the main indices of alpha diversity, including the Chao1 index, Shannon index, and Simpson index, to evaluate the abundance and diversity of microorganisms in different communities. Beta diversity analysis was used to compare the differences in microbial communities; principal coordinate analysis (PCoA) was used to show the differences in community structure between the two groups. The smaller the sample distance was, the more similar the species composition structure; the larger the sample distance was, the greater the community difference. The community differences were analyzed by Adonis; the smaller the *P* value was, the more significant the differences between the groups were. QIIME2 software was employed to estimate the composition of the two groups of samples at six taxonomic levels: phylum, class, order, family, genus, and species. Linear discriminant analysis (LDA) effect size (LEfSe) was performed using the default parameters to identify differentially abundant taxa across the two groups, and the threshold value of the LDA score was set to 4 to find the biomarkers with significant differences.

### Statistical Analysis

SPSS version 24.0 statistical software was used for all statistical analyses (SPSS, Inc). Measurement data with normal distribution were presented with mean ± standard deviation, and the unpaired Student’s t-test was used for between-group comparisons. The chi-square test was used to compare the data of categorical variables, while Fisher’s exact test was performed to compare the differences with the expected count 5 or fewer individuals between the two groups. The non-normal distribution data were represented by the median *M (P25, P75)*. Considering that the sample size was small and the data might not conform to a normal distribution, the Wilcoxon rank sum test was used to compare the differences between the two groups. A *P* value < 0.05 represented statistical significance.

## Results

### The Basic Characteristics of the Patients

The age distribution of the two groups was as follows: 39–58 years (median: 48 years; N group) and 34–72 years (median: 56 years; EC group). There was no significant difference in age, BMI, parity, or comorbidity between the two groups (all *P* > 0.05).

In the EC group, there were 12, 15, and 5 cases of high, middle, and low differentiation, respectively. The surgical pathological staging was as follows: stage IA (19 cases), stage IB (1 case), stage IIIA (1 case), stage IIIC1 (7 cases), and stage IIIC2 (4 cases). The lymph node metastasis statuses were as follows: metastasis (11 cases), no metastasis (21 cases). The clinicopathological characteristics of participants are shown in Table 1. All the patients with endometrial cancer underwent comprehensive staging surgery. Also, all the patients had primary lesions and were not treated with preoperative radiotherapy or chemotherapy.

**Table 1:** Clinicopathological characteristics of participants

Characteristics	Normal endometrium (n = 33)	Endometrial cancer (n = 32)	*P*
Age (median, years)	48 (range 39–58)	56 (range 34–72)	0.144
BMI (kg/m^2^)	24.83±4.02	25.59±3.33	0.412
Parity, n (%) ^a^			1.000
0 para	2 (6.06)	1 (3.13)	
≥1 para	31 (93.94)	31 (96.87)	
Comorbidity, n (%)			
Diabetes mellitus ^a^	2 (6.06)	5 (15.63)	0.285
Hypertension	6 (18.18)	9 (28.13)	0.341
Hyperlipidemia	10 (30.30)	8 (25.00)	0.633
FIGO stage, n (%)			
I	-	21 (65.63)	
II	-	0 (0)	
III	-	12 (37.50)	
IV	-	0 (0)	
Differentiation, n (%)			
Well		12 (37.50)	
Moderate	-	15 (46.88)	
Poor	-	5 (15.62)	
LN metastasis, n (%)			
No	-	21 (65.63)	
Yes	-	11 (34.38)	

^a^ Fisher’s exact test was used because contingency table contained expected counts ≤5

### Characteristics of OTUs

**Fig. 1:**
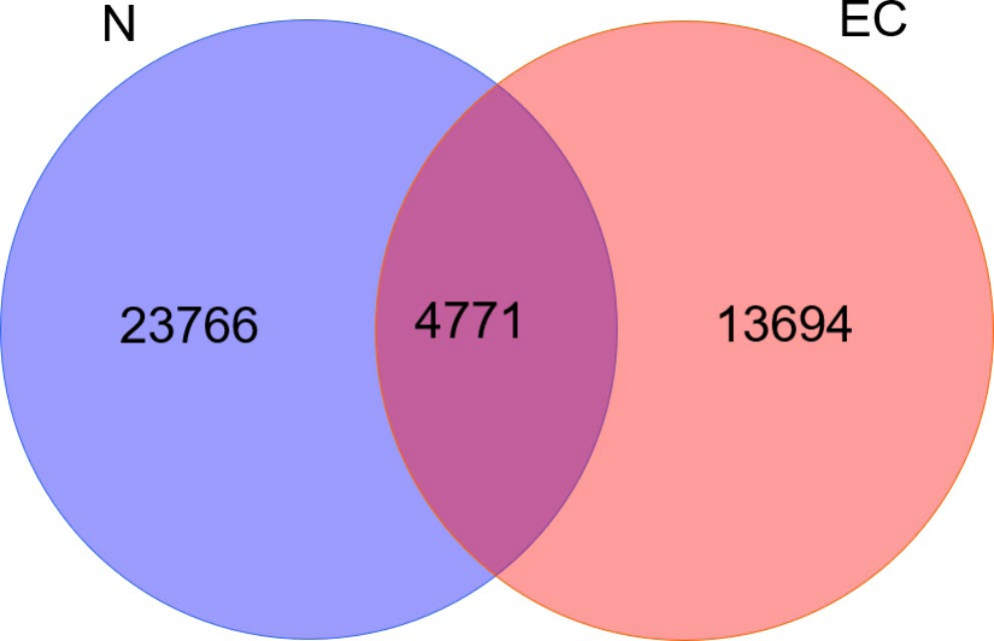
Venn diagram. Venn diagram of operational taxonomic units (OTUs) showed the number of common and unique OTUs, and the similarity and overlap of OTUs between the two groups. N, control group; EC, endometrial cancer group

The gut microbiota composition was investigated by analyzing the sequences of the V3-V4 variable region of the 16S rRNA gene. Sequences with more than 97% similarity were clustered into OTUs. A Venn diagram showed that the total numbers of OTUs in the N and EC groups were 28,537 and 18,465, respectively. The total number of OTUs in the two groups was 42,231, of which the number of OTUs shared by the two groups was 4771, accounting for 11.30% of the total number (Fig. 1).

### Alpha Diversity Analysis of Gut Microbiota in the EC Group and the Control Group

**Fig. 2:**
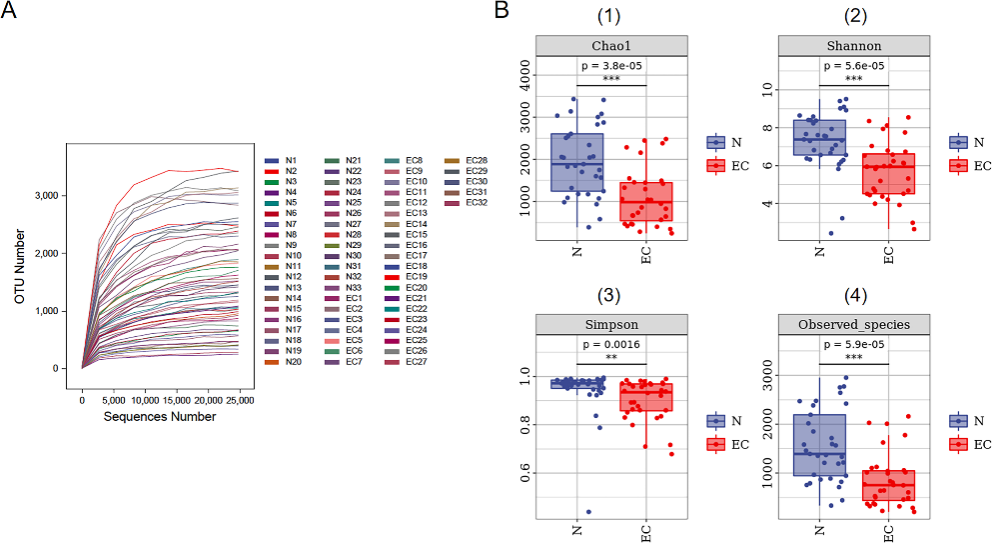
Difference analysis of alpha diversity of gut microbiota in the Control group and EC group. (**A**) Rarefaction curves. **(B)** The alpha diversity between the two groups calculated using four different parameters: (1) Chao1 index, (2) Shannon index, (3) Simpson index, and (4) Observed species. **P < 0.01, ***P < 0.001. N, control group; EC, endometrial cancer group

The rarefaction curve showed that with the increase of the sample sequencing, the number of new OTUs tended to be stable, and the curves were flat, indicating that the sequencing depth of all samples could capture the microbial diversity (Fig. 2A). To evaluate the abundance and diversity of bacteria in the gut microbiome, we calculated the alpha diversity of all fecal samples from all groups using the Chao1, Shannon, Simpson, and Observed species indices. The results showed that the diversity of the gut microbiota was significantly reduced in patients with endometrial cancer vs. healthy controls (*P* < 0.01) (Fig. 2B).

### Beta Diversity Analysis of Gut Microbiota in the EC Group and the Control Group

**Fig. 3:**
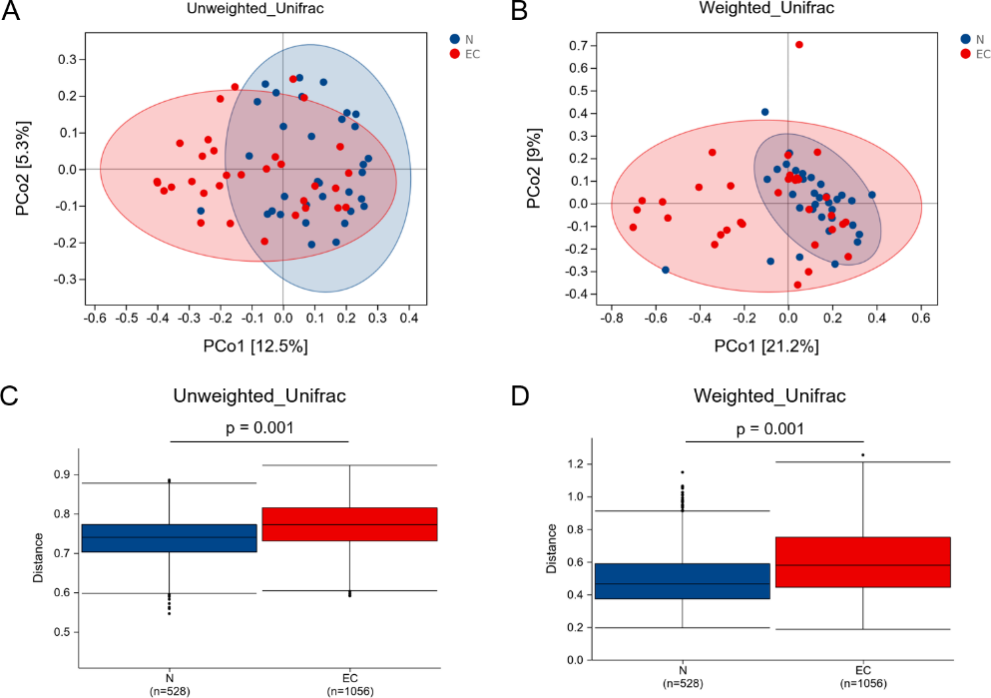
Difference analysis of beta diversity of gut microbiota in the Control group and EC group. (**A**) PCoA analysis based on unweighted UniFrac distance. **(B)** PCoA analysis based on weighted UniFrac distance. **(C)** Box diagram based on Unweighted UniFrac beta diversity. ***P = 0.001. **(D)** Box diagram based on weighted UniFrac beta diversity. ***P = 0.001. N, control group; EC, endometrial cancer group

We performed PCoA based on the unweighted UniFrac distance and the weighted UniFrac distance (Fig. 3A and B). According to the results of Adonis analysis, there was a significant difference in the distribution of microbiota between the two groups (*P* = 0.001, Fig. 3C and D). This is the first study to report the above changes in the alpha diversity and beta diversity of the gut microbiota in endometrial cancer patients, which are different from those in healthy controls.

### Gut Microbial Compositions of the EC Group and the Control Group

**Fig. 4:**
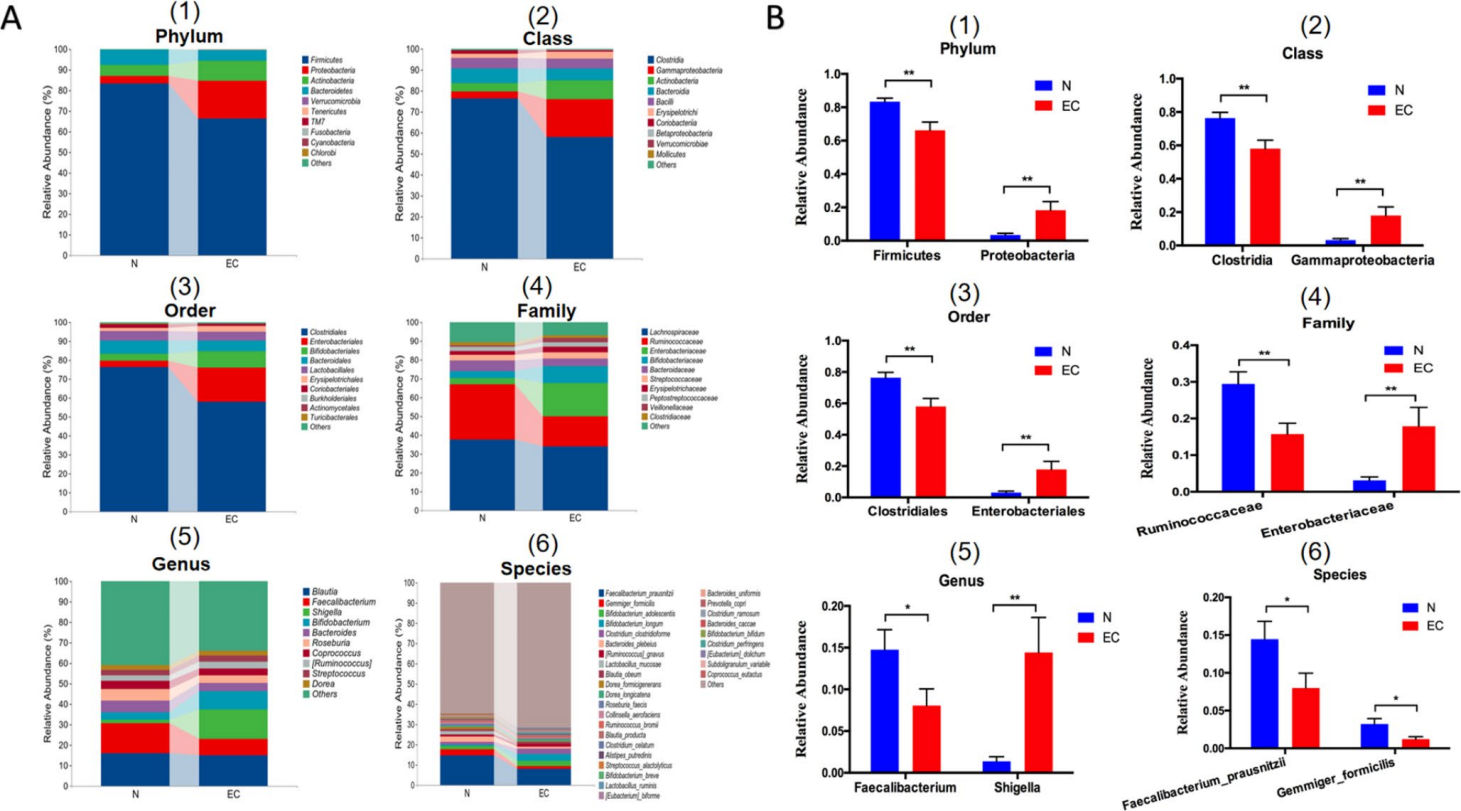
Composition of the gut microbiota and the analysis of species differences at the phylum, class, order, family, genus and species levels between the two groups. (**A1, B1**) Bar charts and the analysis of species differences at the phylum level. **P < 0.01. **(A2, B2)** Bar charts and the analysis of species differences at the class level. **P < 0.01. **(A3, B3)** Bar charts and the analysis of species differences at the order level. **P < 0.01. **(A4, B4)** Bar charts and the analysis of species differences at the family level. **P < 0.01. **(A5, B5)** Bar charts and the analysis of species differences at the genus level. *P < 0.05, **P < 0.01. **(A6, B6)** Bar charts and the analysis of species differences at the species level. *P < 0.05. N, control group; EC, endometrial cancer group

We performed clustering analysis between the EC and control groups at the phylum, class, order, family, genus, and species levels. From the results of the phylum-level analysis, *Firmicutes, Proteobacteria, Actinobacteria*, and *Bacteroidetes* were the most important phyla in the two groups of samples, accounting for more than 99% of the total. The abundance of *Firmicutes* (*P* = 0.0018) decreased and that of *Proteobacteria* (*P* = 0.0055) increased in the patients with endometrial cancer vs. healthy controls (**Fig. **4A1, 4B1).

At the class-level analysis, the abundance of *Clostridia* (*P* = 0.0039) decreased and that of *Gammaproteobacteria* (*P* = 0.0057) increased in the EC group vs. healthy controls (**Fig. **4A2, 4B2).

At the order level, the abundance of *Clostridiales* (*P* = 0.0038) decreased and that of *Enterobacteriales* (*P* = 0.0057) increased in the EC group vs. healthy controls (**Fig. **4A3, 4B3).

At the family level, the abundance of *Ruminococcaceae* (*P* = 0.003) decreased and that of *Enterobacteriaceae* (*P* = 0.0057) increased in the EC group vs. healthy controls (**Fig. **4A4, 4B4).

At the genus level, the abundance of *Faecalibacterium* (*P* = 0.0366) decreased and that of *Shigella* (*P* = 0.0027) increased among the EC patients vs. healthy controls (**Fig. **4A5, 4B5).

At the species level, the abundance of *Faecalibacterium prausnitzii* (*F. prausnitzii*) (*P* = 0.041) and *Gemmiger formicilis* (*G. formicilis)* (*P* = 0.0132) in the EC group significantly decreased (**Fig. **4A6, 4B6). Moreover, the abundance of individual bacterial species, such as *Bifidobacterium adolescentis*, increased in the EC group, but the increase was not statistically significant. The above findings of this study were different from those of the previous research. In this study, a more detailed clustering analysis was conducted from six different levels.

### LEfSe Analysis of the Gut Microbiota Between the EC Group and the Control Group

**Fig. 5:**
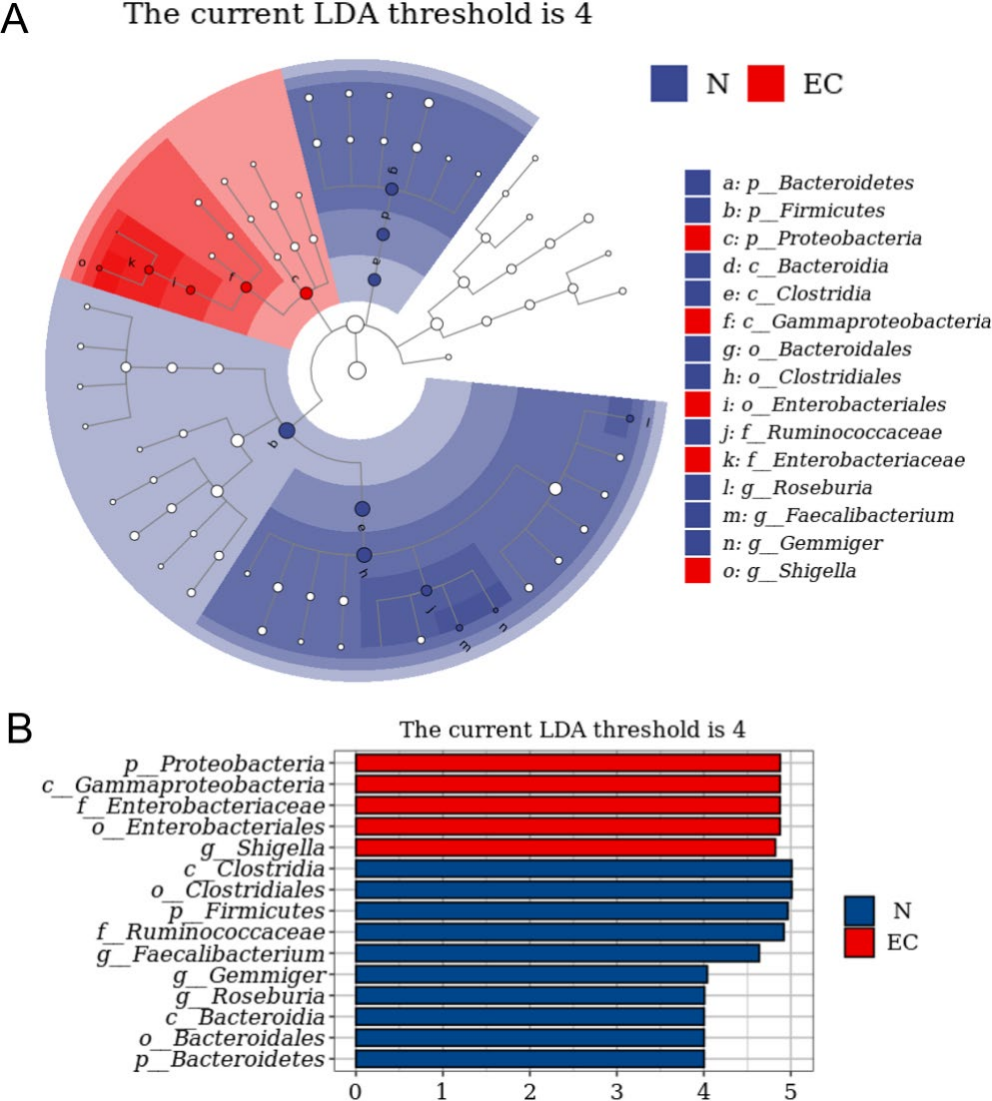
LEfSe analysis of the gut microbiota between the two groups. (**A**) The cladogram showing differentially abundant taxa between the N group and EC group (LDA score > 4.0). **(B)** Histogram of the distribution of the LDA score between the two groups. Each small circle at different taxonomic levels represents a taxonomy at that level, and the diameter of the small circle is proportional to the relative abundance. N, control group; EC, endometrial cancer group

LEfSe is commonly used for the discovery and interpretation of high-dimensional potential biomarkers. The gut microbiome of the two groups was compared by LEfSe analysis to find biomarkers with significant differences (Fig. 5). The histogram of the LDA score distribution showed the species with LDA scores greater than the threshold, i.e., the biomarker with a significant difference between the groups. The species with significant differences in abundance in the different groups are shown, and the histogram length represents the effect size of different species. In the cladogram, each small circle at various taxonomic levels represents taxonomy at that level, and the diameter of the small circle is proportional to the relative abundance. The results showed that the predominant bacteria in the N group were *Firmicutes*, *Bacteroidetes*, *Clostridia*, *Bacteroidia*, *Clostridiales*, *Bacteroidales*, *Ruminococcaceae*, *Faecalibacterium*, *Gemmiger*, and *Roseburia*, while the predominant microbiota in the EC group were *Proteobacteria*, *Gammaproteobacteria*, *Enterobacteriales*, *Enterobacteriaceae*, and *Shigella*. These results were consistent with those of the differential microbiota analysis.

## Discussion

Thus far, existing studies have mainly focused on examining the effect of endometrial microflora on endometrial malignancies, including endometrial cancer, while the role of the gut microbiome and endometrial cancer is still not fully understood [22]. In this study, we implied that alteration in the gut microbioma might be an indirect factor in the development of endometrial cancer. Several previous studies have found that an imbalance in the gut microbiome has a vital role in the occurrence of obesity, PCOS, and abnormal estrogen metabolism, which are considered strong risk factors for endometrial malignancies [19, 23]. Studies on the characteristics of gut microbiota in patients with endometrial cancer are rare, with only individual reports, and the number of cases included in the study is also very small. This study included more and different cases for research, which helps to further elucidate the relationship between intestinal microbiota dysbiosis and endometrial cancer. This study assessed intestinal microbiome differences between patients with endometrial cancer and healthy controls on the relatively advanced Illumina NovaSeq platform. A more detailed clustering analysis was conducted from six different levels: phylum, class, order, family, genus, and species and there are new findings in this study. The results of 16S rRNA gene sequencing showed that the alpha diversity of the gut microbiota was significantly reduced, and the distribution of bacteria was significantly different in patients with endometrial cancer. *Firmicutes*, *Proteobacteria*, *Actinobacteria*, and *Bacteroidetes* were the most important phyla in the two groups of samples, which was consistent with the general characteristics of the human gut microbiota [10]. However, the abundance of *Firmicutes*, *Clostridia*, *Clostridiales*, *Ruminococcaceae, Faecalibacterium*, and *Gemmiger_formicis* significantly decreased, while that of *Proteobacteria*, *Gammaproteobacteria*, *Enterobacteriales*, *Enterobacteriaceae* and *Shigella* significantly increased in the EC group compared with the control group. Moreover, LEfSe analysis showed that the predominant microbiota of the two groups was significantly different. The predominant microbiota of the N group was *Firmicutes*, *Bacteroidetes*, *Clostridia*, etc., while the predominant microbiota of the EC group was *Proteobacteria*, *Gammaproteobacteria*, etc. These results were consistent with the results of the differential microbiota analysis. The above findings demonstrated that the abundance, diversity, and predominant microbiota of the gut microbiome in patients with endometrial cancer exhibited significant changes, which may be closely related to the tumorigenesis or progression of endometrial cancer.

In recent years, there have been reports on the role of gut microbiota in malignant tumors. Abdulamir et al. [24] found that an increase in pathogenic *Streptococcus bovis* in the colon was associated with colon cancer. Transplanting feces from colorectal cancer patients into sterile mice can cause malignant tumor lesions and epigenetic changes in DNA methylation [25]. Wang et al. found that the composition and diversity of the gut microbiota in patients with cervical cancer significantly changed compared with that in the healthy population. The α-diversity of the gut microbiota showed an upward trend, with a significantly higher proportion of the phylum *Proteobacteria* [26]. There have also been reports on the mechanism through which the gut microbiota affects the occurrence of malignant tumors. Studies have found that the induction of tumors by disordered gut microbiota may be associated with the mediated chronic inflammatory response, immune abnormalities, the production of toxic substances, and biological behaviors such as tumor cell proliferation and apoptosis [27]. It has been well demonstrated that specific bacteria or adverse biological bacterial communities cause epithelial barrier failure, immune dysfunction, and genotoxicity, thus creating a tumor-permissive microenvironment [28, 29].

The present study showed that the composition of the gut microbiota in patients with endometrial cancer was disordered, the diversity of the microbiota was reduced, and the distribution was abnormal. This imbalance in the gut microbiota may have a tumor-promoting role in endometrial cancer through similar mechanisms reported above. In addition, the imbalance in the gut microbiota may affect the occurrence and progression of endometrial cancer by changing the metabolic function and estrogen level and then further promoting the occurrence of high-risk factors such as obesity and diabetes. However, the specific molecular regulation mechanism is not clear. At the species level, the abundance of *F. prausnitzii* (*P* = 0.041) and *G. formicilis* (*P* = 0.0132) in the EC group significantly decreased (**Fig. **4A6, 4B6). Moreover, the abundance of individual bacterial species, such as *Bifidobacterium adolescentis (B. adolescentis)*, increased in the EC group, but the increase was not statistically significant. *F. prausnitzii* is one of the main butyrate producers in the intestine that protects the colon from cancer and inflammatory bowel diseases by reducing inflammation [30]. Moreover, *c*olonization by Firmicutes, specifically *F. prausnitzii* and *G. formicilis* is associated with anti-cancer response and immune-related colitis [31]. Bifidobacterium species are usually considered nonpathogenic and may be found in the human intestine, vagina, and oral cavity.

The gut microbiota is dynamic and adjustable throughout a person’s life and can be affected by many factors, such as the host’s diet, environment, physical health status, and medication use. The results of this study suggest that regulating the composition of the *gut microbiota* and restoring intestinal microecological balance may be a new strategy for preventing and treating endometrial cancer. Clinically, the imbalance in the gut microbiota can be adjusted through the rational use of antibiotics, microbial preparations, diet regulation, fecal bacteria transplantation, and other measures.

There are some limitations in this study. First, this study has a small sample size. Second, we did not stratify patients based on obesity, hypertension, and diabetes. Third, we found the characteristics of the changes in the gut microbiota in patients with endometrial cancer, but the specific metabolic function of these gut microbiota and the direct relationship with the development and progression of endometrial cancer remain unclear. In our next study, we plan to further screen patients based on obesity, age, etc., and asses possible translocation of gut bacteria in the endometrium, which will further clarify the relationship between gut microbiota and cancer. In addition, we plan to select the differential microbioma and conduct separate studies on the gene function and metabolite detection of the microbioma, explore the relevant signaling pathways, and further explore the molecular mechanisms.

## Conclusion

Through high-throughput 16S rRNA gene sequencing, this study revealed that the diversity, distribution and composition, and predominant microbiota of the gut microbiota in patients with endometrial cancer significantly differed from those in a normal endometrial population at different taxonomic levels. In addition, potential biomarker microbial species (including *F. prausnitzii* and *G. formicilis*) were identified. These results suggest that regulating the composition of the gut microbiota may be a new strategy for preventing and treating endometrial cancer.

## Electronic Supplementary Material

Below is the link to the electronic supplementary material.

Supplementary Material 1

Supplementary Material 2

## Data Availability

The raw 16S rRNA gene sequences of 65 fecal samples were deposited in NCBI Sequence Read Archive (SRA) database with the accession number PRJNA897842.
