# Supplementary material for: Gut Microbiome Dysbiosis in Patients with Endometrial Cancer vs. Healthy Controls Based on 16S rRNA Gene Sequencing
Source: Curr Microbiol. 2023 Jun 9;80(8):239. doi: 10.1007/s00284-023-03361-6 (PMC10256650; doi:10.1007/s00284-023-03361-6)
Supplement: Supplementary file 1 — Supplementary Material 1 [file 284_2023_3361_MOESM1_ESM.pdf]

# Current Microbiology

**AUTHOR'S CHECKLIST FOR MANUSCRIPT SUBMISSION**

**A copy of this checklist should accompany each submitted paper.**

**Your manuscript must be prepared according to the Instructions for Authors which are available online and accessible[here](#).**

**General**

☐√ **At least the corresponding author has read carefully the Instruction for Authors guide.**

☐√ All authors have read the submitted version of the paper AND have approved its Submission.

☐√ All authors' names have been provided as 'full first name, (middle initial), full last name'

☐√ All authors have double checked their affiliation (with precise location).

☐√ The contact information for the corresponding author(s) is as follows:

- Provided an Institutional email/ORCID number whenever possible
- Institutional phone number

☐√ All text, including abstract, tables, references, footnotes and figure legends, is double- spaced printed. All lines and pages are numbered.

**Compliance with ethical standards**

☐√ Authors' contribution statement has been included.

☐√ A disclosure of potential conflict of interest has been included.

☐√ Ethical approval for research involving human participants and/or animals: if applicable

☐ Informed consent has been provided: if applicable (human participants)

**Abstract**

☐√ The abstract is clear and concise and summarizes the content of the paper without presenting extensive experimental details.

- Length (250 words max)
- Structure: one paragraph without subheadings
- Keywords (5-7)

**Manuscript**

☐√ The English language usage has been checked by a fluent English speaker and/or a professional language editing service.

☐√ The length of the manuscript is according to Instruction for authors taking into account the article type.

☐√ Abbreviations are defined at first mentioning.

☐√ Figures have been submitted electronically separately from the main manuscript file, at high resolution and preferentially in TIFF or EPS.

☐√ Figure captions summarize the result or major of the data you are presenting in the figure with a brief description of the methods necessary to understand the figure without having to refer to the main text.

☐√ The submission has no more than five main figures with up to four panels each (Original paper) or up to two figures with up to four panels each (Short Communications).
☐√ Reference formatting is according to the formatting outlined in the instructions for authors.
☐√ The number of references is according to the article type.

**Data**
☐√ Raw data/original images of uncropped and non-adjusted gels/blots should be available to be provided as supporting material upon request.
☐√ The sequence accession number(s) have been provided at the end of Material & Methods section.

**New taxa**
☐ The type strains for new taxa have been deposited in at least two different culture collections
☐Full (required for prokaryotes) or draft genome sequencing data is provided.

for fungi see:https://doi.org/10.1186/s43008-021-00063-1
for prokaryotes see:https://doi.org/10.1099/ijsem.0.000778

**Microbial isolates**
☐Microbial isolates should be correctly identified and characterized using molecular methods.
☐In case of 16S rRNA gene sequence comparison (more than 1000 nt) is mandatory between prokaryotic isolates and type strains of validly named species.
☐ The isolate identification was performed following the Submission guidelines (Microorganism Identification section) and recommendations published in thisEditorial.

**Statistics**
☐√ A clear experimental design with the treatments and replicates clearly described has been included.
☐√ The F values, the degrees of freedom and the precise p values obtained have been calculated.
☐√ The assumptions of the statistical test have been tested.
☐√ Statistical information (number of replicates, asterisks denoting P-values, statistical tests) has been provided.

**Suggested Reviewers**
☐√ Suggested reviewers are from different countries and different institutions than the submitting authors.
☐√ The reviewer candidates have a verifiable email address and affiliation.

**Springer Nature Editorial Policies**
☐√ The manuscript presented adheres to the Editorial Policies of Springer Nature.

**All policies, also including our preprint policy are accessible here:**
**https://www.springernature.com/gp/policies/editorial-policies**
