# Supplementary material for: Gut Microbiome Dysbiosis in Patients with Endometrial Cancer vs. Healthy Controls Based on 16S rRNA Gene Sequencing
Source: Curr Microbiol. 2023 Jun 9;80(8):239. doi: 10.1007/s00284-023-03361-6 (PMC10256650; doi:10.1007/s00284-023-03361-6)
Supplement: Supplementary file 2 — Supplementary Material 2 [file 284_2023_3361_MOESM2_ESM.docx]

**Cover letter**

Dear editor:

Here is our paper submitted to Current Microbiology.

The title: Gut microbiome dysbiosis in patients with endometrial cancer vs. healthy controls based on 16S rRNA gene sequencing

The authors: Yue Li, Geng Liu, Runqi Gong, Yong Xi.

In this paper, we found that the alpha diversity of the gut microbiota was significantly reduced in the endometrial cancer patients. There was a significant difference in the distribution of flora between normal endometrial population and endometrial cancer patients. The predominant microbiota of the endometrial cancer patients was Proteobacteria, Gammaproteobacteria, Enterobacteriales, Enterobacteriaceae and Shigella. Adjusting the composition of the gut microbiota may be a new strategy for preventing and treating endometrial cancer.

All authors have contributed to, read and approved the final manuscript for submission.

The authors claim that none of the material in the paper has been published or is under consideration for publication elsewhere.

All authors have no conflict of interest.

This study was approved by the Ethics Committee of Dalian Municipal Women and Children's Medical Center (Group). The written informed consent has been obtained.

Thank you very much for consideration!

Sincerely yours,

Dr. Yong Xi
